# The silent world of assisted reproduction: A qualitative account of communication between doctors and patients undergoing in vitro fertilisation in Australia

**DOI:** 10.1111/hex.13839

**Published:** 2023-08-04

**Authors:** Louis Taffs, Ian Kerridge, Wendy Lipworth

**Affiliations:** ^1^ Sydney Health Ethics, Faculty of Medicine and Health The University of Sydney Camperdown New South Wales Australia; ^2^ Haematology Department Royal North Shore Hospital St Leonards New South Wales Australia; ^3^ Department of Philosophy Macquarie University Macquarie Park New South Wales Australia

**Keywords:** ART, consent, corporatisation, ethics, IVF, patient–doctor relationship, treatment burden

## Abstract

**Context:**

In vitro fertilisation (IVF) is now a common assisted reproductive technology (ART) procedure globally, with 8 million children alive today having been conceived utilising IVF. For many patients, IVF is a difficult experience with many discontinuing treatment because of emotional, relationship and financial stress, or intolerable physical side effects of hormone treatments.

**Design and Participants:**

A qualitative study, in which 31 professionals and 25 patients from the ART sector in Australia were interviewed. The interviews were analysed using codebook thematic analysis.

**Results:**

Our data indicates there are ‘silences’ within the therapeutic relationship of IVF, which may limit the capacity for patients to prepare emotionally, financially, or medically for the procedure, and may contribute to psychological distress and dissatisfaction with care. These ‘silences’ include what the patient ‘is not told’ by their clinician or ‘does not hear’ and what the patient feels they ‘cannot say’.

**Discussion:**

Drawing upon the work of Jay Katz, Charis Thompson, and Miles Little on ‘silences’ and performance in clinical practice, we argue that although IVF is a complex and multifaceted procedure that is often conducted in a commercial setting, the clinical and therapeutic relationship between doctor and patient remains pivotal to the experiences of patients. The ‘silences’ within this relationship may impact negatively on decision‐making, and on the delivery and experience of care.

**Conclusions:**

Careful attention to the realities of IVF treatment in the clinic room (and awareness of the performances that hide them) should allow for more present and compassionate care. Such care may leave patients more satisfied with their experience and their choices, regardless of treatment outcomes.

**Patient or Public Contribution:**

This article draws on interviews with patients who had undergone or were currently undergoing IVF, as well as a range of representatives from the ART community (including reproductive medicine specialists, general practitioners, fertility nurses, counsellors, administrators in ART businesses and embryologists).

## 1 INTRODUCTION

Assisted reproductive technology (ART) describes a range of medical interventions used to aid couples and individuals to conceive. One of the most commonly utilised interventions is in vitro fertilisation (IVF) (literally ‘fertilisation in glass’) which refers to the ex vivo fertilisation and subsequent implantation of embryos generated from oocytes that have been retrieved from the ovaries after hormonal stimulation. The first successful pregnancy using IVF was in 1978.^1^ There are 8 million people alive today who have been conceived using IVF and in countries with well‐developed health systems, as many as 1 in 20 registered births result from IVF.^2^

IVF poses an obvious solution to many causes of infertility, such as nonpatent fallopian tubes or poor sperm motility. However, IVF is associated with well‐recognised side effects related to ovarian stimulation including nausea, abdominal tenderness and bloating and, in more severe cases, ovarian hyperstimulation syndrome (OHSS) (this occurs in 3%–10% of IVF patients and can cause abdominal pain, blood clotting, weight gain, shortness of breath, organ failure and rarely, death).^3^ The IVF process is also often psychologically challenging,^4^, ^5^ particularly during periods of waiting to find out if retrieval or implantation has been successful.^6^

In this article, we report the results of an interview study funded by the Australian *National Health and Medical Research Council* that was conducted with a range of professionals working within the ART industry in Australia, and with patients who had received or were receiving IVF. Although the study was not solely focused on elucidating the psychological dimensions of ART, it emerged from the data that both professionals and patients are highly alert to these dimensions. Here, we report on these findings and then interpret the themes that emerged by drawing on the work of Katz,^7^ Thomason^8^ and Little.^9^ This reveals how the experience of delivering and receiving IVF represents a conversational dance that nudges *both* doctor and patient away from frank discussions about the realities of IVF treatment, leaving patients feeling confused and disempowered.

## 2 METHODOLOGY

### 2.1 Recruitment

#### 2.1.1 Professional participants

Thirty‐one professional participants, including ART specialists, nurses, counsellors, embryologists, clinic managers, referring practitioners and regulators, were recruited via the professional body representing ART specialists in Australia (the Fertility Society of Australia and New Zealand), the professional contacts of the research team and snowball sampling. To ensure that no important themes were missed, we recruited participants who worked in academia, public facilities, standalone private clinics and clinics that are part of larger corporate organisations. We also made efforts to include older and younger clinicians. Second (follow‐up) interviews were conducted with 16 of the participants to further explore emergent issues (Table 1).

**Table 1:** Demographic and occupational characteristics of the 31 ART industry experts.

Gender			
Women		19	
Men		12	
Total		31	

Age		**Mean (years)**	**Range (years)**
Women		50.0	32–66
Men		62.1	39–75
Total		54.4	32–75

Main current role	**Total**	**Female**	**Male**
Patient advocate	2	1	1
Regulator	2	1	1
Manager	5	2	3
Fertility specialist	11	6	5
Nurse	2	2	–
Counsellor	2	2	–
Scientists	3	2	1
Embryologists and reproductive biologists	2	2	–
Referring general practitioner	2	2	–

The sector of main current/most recent role if retired			
Public clinic	–		
Commercial clinic—corporate chain—listed	11		
Commercial clinic—corporate chain—privately held	4		
Commercial clinic—standalone	6		
Academia	3		
Independent, other agency, e.g.	5		
General practice	2		

Country, state or territory of current/most recent role			
NZ	1		
Australia, nationwide role	1		
NSW	11		
VIC	9		
SA	5		
QLD	3		
WA	1		
ACT	–		
NT	–		
TAS	–		

Practice setting			
Urban	25		
Mixed	4		
Rural	–		
Not stated	1		
Not applicable	1		

Abbreviation: ART, assisted reproductive technology.

#### 2.1.2 Patient participants

Twenty‐five patients were recruited via social media (Facebook and Twitter) and press releases and advertisements in regional newspapers. The patient participants sought ART for a variety of reasons and were at various stages of the ART process. They were from both metropolitan and regional/rural areas and were in a variety of relationship circumstances (single women, same‐sex couples and mixed‐sex couples) (Table 2).

**Table 2:** Demographic characteristics of the 25 ART patient participants.

Age (years)	
Mean	37
Range	26–50
Setting	
Metropolitan	13
Rural and regional	12
Family structure	
Single	9
Same‐sex couple	4
Mixed‐sex couple	12
States, territories and countries represented in the sample	
NZ	1
NSW	6
Victoria	11
Queensland	2
South Australia	–
Tasmania	4
WA	1
Northern Territory	–
ACT	–
Occupational categories represented in the sample	
Education	2
Policy and research	5
Law	1
Insurance	1
Public service	1
Administration	2
Allied health, public health and health sciences	4
Medical	5

Abbreviation: ART, assisted reproductive technology.

### 2.2 Analysis

Interviews were conducted between September 2020 and September 2021 via videoconference (Zoom) and lasted for an average of 79 min. The interviews were recorded and transcribed using an online transcription service, Digital and Audio Transcription Service.

The data were initially analysed by two researchers using codebook thematic analysis, which involves developing a coding framework consisting of domain summaries of topics.^10^ These were then organised into categories aligned with the study research questions. A more focused thematic analysis was conducted to further explore themes of interest, including those that cut across domains. Here, we report on our analysis of themes that pertained to patient experience and discursive characteristics of the doctor–patient relationship, and the ‘silences’ that characterise these relationships.

## 3 RESULTS

### 3.1 What the patient ‘is not told’

It is now widely assumed that decisions about health care should be made by both patient and doctor after the provision of adequate information and through a process of shared decision‐making. For a patient's decisions to be ethically and legally valid, they must have sufficient capacity, be provided with sufficient information, have some degree of understanding or appreciation of that information relevant to their situation, and make decisions freely and voluntarily and authorise clinicians to proceed. Despite the fact that these elements of consent are widely recognised in law, ethics and practice, a number of participants in our study reported that there were inadequacies in the information provided to patients.

Interviewer: ‘And so, throughout that, did you feel like you were in a good position to accept or refuse some of the treatments that were being suggested to you?’

Consumer 7: ‘No’.

Interviewer: ‘No? And was there any discussion of, you know, alternative options or going for second opinions?’

Consumer 7: ‘No’.

Interviewer: ‘And was it clear where you could go to find out more information about some of the treatments that were being suggested?’

Consumer 7: ‘Um, no. You just have such trust in your doctor…

…And so, at no point in that conversation was it, we're going to do it because of this, and this is going to be the cost, and this is what the recovery is going to be like. There was none of that. So, all of that then is a shock thereafter. So that full disclosure of what that procedure was and what it would affect timeline‐wise wasn't there.’

Importantly, even those patients who believed they had been given sufficient information about the technical and physical aspects of treatment reported feeling taken aback by the less tangible realities of the treatment. There were two reasons offered for this: First, some experiences simply cannot be communicated until one experience for themselves.

‘But yeah, I – I guess, I was – I was prepared for the injections…But I wasn't prepared for lots of other things and I wasn't prepared for the emotional journey… And I – I don't know – I – I guess it's one of those things as – as – as a doctor or nurse or somebody could say to you, IVF is emotional. IVF is emotional, but until you're in it, you don't realise how emotional it will be.’ Consumer 6

One fertility counsellor recognises this, forewarns her patients that they may be surprised by some aspects of treatment, and invites them to contact her throughout the course of treatment.

‘I would make myself available for ten or 15‐min appointments when people were commencing their first IVF cycle. And it was really a, ‘Hi, I'm the counsellor. You're probably fine right now, and don't see why you might need to talk to someone like me but I'm just letting you know that you've probably got a bit of a hard road ahead. These are some of the things to expect. These are some of the things to take care of’. And I would quite often have people find me two or three years down the track and say, ‘I didn't quite understand at the time, what it was going to be like’. Expert 13 (Counsellor)

The second reason that patients who are given sufficient information may nonetheless find themselves unprepared for the realities of treatment is that information has been framed in particular ways. For example, certain realities might be downplayed by clinicians who want to reassure patients and keep them hopeful and motivated to continue treatment. Although participants did not admit explicitly to providing misleading information, several noted how important it is to keep patients motivated, and it seems likely that communication would have been affected by this aim:

‘Yeah. So the thing is, well, IVF is really tough. So if you think they're going to get there in the end but, in order for them to get them there, they're not, they're going to have to do a few cycles, the key then is to keep them on the treadmill because you know they'll get there because there's so many things where they could step off. And if they step off for a year and then jump back on, it's going to be a hell of a lot harder then.’ Expert 18 (IVF Clinician)

### 3.2 What the patient ‘does not hear’

While the way in which information is communicated is undoubtedly crucial, patients may also not be willing or able to ‘hear’ some information that is provided to them.

‘I think it goes both ways. I think that specialists need to get better at telling us yeah, the negative. But I think patients also need to own not listening to the things that they don't want to hear…And so I think a lot of people go into the process, not knowing what it is that they're signing up for…they know it's injections and that you end up with you end up with a baby… not the ups and downs that come in between.’ Consumer 6

Patients' unwillingness to ‘hear’ certain things may at times relate to information they have obtained before seeking expert advice. Many fertility clinicians spoke about the increasing number of patients who arrive at the clinic armed with the information they had collected online or from their peers and friends and request particular interventions. This creates a moral dilemma for clinicians if they do not agree that the intervention is in the patient's best interest and may impact negatively on communication if patients do not want to listen to the clinician's reasoning.

‘So look, I think the patients – because there's plenty of choices out there – I think [if] the patients want that treatment, they'll find themselves a doctor who'll provide it. They won't necessarily engage with the doctor who wants to talk about the evidence.’ Expert 11 (IVF Clinician)

### 3.3 What the patient feels they ‘cannot say’

In an ideal world, the process of informed consent involves more than a doctor reading from a list of known risks to a procedure and a patient signing a waiver, protecting the doctor from litigation. It is a conversation about goals, values, and what aspects of various treatments may either facilitate or compromise those for the patient. For this reason, it needs to be a conversation, in which both the doctor and the patient are permitted to voice their concerns and share their mutual knowledge to come to a shared decision. However, in the world of IVF, it can be difficult for the patient to have a voice in this conversation.

‘I think that I come from a pretty high level of health literacy and I found it really difficult and not only difficult to talk about but difficult to get information.’ Consumer 1

For some, it was because they feel they have chosen to be there and have no ‘right’ to complain or reason to refuse.

‘And you know, let's not forget it's elective to do IVF. So it's not like, you know, you're still, you're going through surgical procedures and medication, yet you feel, I felt, always felt like I couldn't complain because I was choosing to do that – do that journey. It wasn't like you had cancer or a disease or something.’ Consumer 10

Patients might also be reluctant to express themselves fully because they want to ‘perform’ a particular kind of ‘patient hood’: They might, for example, want to be perceived as being ‘nice’,

‘I like to think I'm a really nice patient and that probably sounds really stupid. But I take great pride in being a very nice person and yeah, being a nice patient to deal with.’ Consumer 15

or they might wish to be perceived as being resilient and ‘normal’.

‘Patients, like I said, they don't cry in front of their fertility clinician, if you get referred to the counsellor it's like you're being sent to the naughty corner. It's that rule again, I must not be doing very well. And, again, I don't think these are deliberate messages. It's the implicit message that sits behind a lot that, ‘I know I'm abnormal if I'm being sent to the counsellor’, because that is not what usually happens.’ Expert 13 (Fertility Counsellor)

This desire to perform (and communicate) in a particular kind of way could stem from a fear that negativity would affect their chances of pregnancy.

‘Again, one of the things that you see in this industry is the sense that – firstly, they have to be positive in order for it to happen and I work a lot on dispelling the myths. I call it the tyranny of positivity because the truth is that actually it is tyrannical. And there is no clinical evidence that you have to be positive in order for this to happen.’ (Expert 9)

It could also stem from a patient's recognition of the power imbalance inherent in the relationship and fear that their doctor will judge and/or reject them if they express any form of distress or are not sufficiently ‘positive’.

‘It's another rule, patients know you don't cry in front of your fertility specialist. That means you're broken. So if I go back to what I was talking about before, about the rules of how patients behave, you'll always look okay in front of your fertility specialist because people are afraid that if they don't, they might be refused care.’ Expert 16 (Fertility Counsellor)

‘I remember just being really quiet, hoping to God that he would just let me keep going [with more cycles of IVF]…I was terrified of him saying, that's it, no more.’ (Consumer 10)

## 4 DISCUSSION

These interviews have illustrated the many ‘silences’ in the doctor–patient relationship, including the words a doctor ‘does not say’ and meanings a patient ‘does not hear’. Furthermore, our data indicate in the world of ART, the voice of the patient is stifled by external and internalised restrictions on honesty and frankness, resulting in patients having things they feel they ‘cannot say’.

### 4.1 Strengths and limitations

The strength of this data is that it provides complementary insights from all stakeholders involved in ART, including experienced ART doctors, nurses, and fertility counsellors and patients. Collectively, the interviews provide a rich account of the experience of delivering and receiving care. The weaknesses of the study are those that characterise all qualitative research: although we endeavoured to include as wide a range of participants as possible, some groups (e.g., male patients or partners) were not recruited; and our findings cannot be assumed to be representative of all stakeholders in even the groups that were included. Participants might also have wanted to present themselves and their experiences in particular ways, which may or may not reflect their ‘true’ views or experiences. Because themes related to silences were emergent and the study was not longitudinal, we were unable to explore nuances such as the different silences that emerge at different points of a patient's journey; however, the fact that these findings emerged without any prompting also adds to their validity, because any statements made were spontaneous.

### 4.2 Theoretical implications

There are many possible explanations for the silences identified in this study. Regarding the theme, ‘what patients are not told’, the most obvious and benign explanation is that doctors worry about inducing unnecessary stress. ART is an emotionally taxing endeavour,^11^ and conversations with doctors about results and prognosis are known to induce stress.^12^ This may, in turn, decrease the likelihood the patient opts for further cycles, or even potentially falls pregnant.^13^ Appreciating this, it is easily understood why doctors would be tempted to withhold potentially distressing information.

To make sense of our findings, we draw on the work of physician, bioethicist, and scholar of law and psychoanalysis Jay Katz. Writing about things that are not said in the clinic or the consulting room, Katz argues the practice of withholding information or presenting it in such a way that obfuscates risks, can at times be less about protecting patients and more about maintaining power in this dyadic relationship. Importantly, according to Katz, this maintenance of power may be well‐intentioned. In his book ‘The Silent World of Doctor and Patient’ (1984), Katz discusses the way in which medical decision‐making has traditionally relied upon patients trusting their physician, relinquishing autonomy, and silently complying with care. In his book, Katz clarifies what he means by ‘silence’.

I do not mean to suggest that physicians have not talked to their patients at all. Of course, they have conversed with patients about all kinds of matters, but they have not, except inadvertently, employed words to invite patients’ participation in sharing the burden of making joint decisions. (p. 3)

With this framework in mind, Katz explores the ethical construction of therapeutic decision‐making and the legal doctrine of informed consent from its classical beginnings. He quotes, for example, the classic Hippocratic text, *Decorum 16*:

Do everything in a calm and orderly manner, concealing most things from the patient while treating him. Give what encouragement is required cheerfully and calmly, diverting his attention from his own circumstances; on one occasion rebuke him harshly and strictly, on another console him with solicitude and attention, revealing nothing of his future or present condition. (p. 4)

These texts, which were drafted at the same time as the Hippocratic oath associate telling the truth with doing harm. Katz writes that this behaviour may have once been necessary, as the tools of the healers of the past were limited and the therapeutic relationship was more foundational to the healing capabilities of a doctor.

In the 20th century, crimes committed against humanity in the name of medicine, changing consumer expectations, and the emergence of medical ethics as a discipline brought to the fore the importance of respecting patients' autonomy.^14^ Over the course of the 20th century, calls for autonomy (particularly in regard to consent and decision‐making) were enshrined in laws around the world. Katz notes, however, that although informed consent and the legal and medical procedures around it were borne out of an honest desire to increase patient autonomy, the practice of it often falls short. He argues that ‘those who are committed to greater patient self‐determination can, if they look hard enough, find inspiration in the common law of informed consent…[but it] is still largely a mirage’.^7^

Katz's work provides a lens through which we can make sense of our data. Our finding that there are many things that the patient ‘is not told’ could represent the continued hesitation that doctors have to reveal too much to the patient, believing that withholding at least some information or framing it in ways that elide certain facts may be in the patient's best interests.

But the accounts provided by participants in our study also align with Katz's concern that patients may feel abandoned and betrayed when the facts and realities of treatment that are concealed eventually come to light. Katz's work and our data also suggest that patient's silences might stem from their own sense of the power imbalance inherent in the relationship and their desire to protect the relationship by being (or at least appearing to be) trusting, compliant and, particularly in this context, sufficiently grateful.

While Katz perceives the doctor–patient relationship as a dyad that is entered into unwillingly by someone who is sick, Charis Thompson in her book ‘Making Parents’ posits that many patients undergoing IVF feel it is elective and therefore perceives themselves as active users of a system. Further to this, and particularly salient in regard to ART, the pressure attached to a potential identity as a parent (and particularly a mother) extends into a person's identity as a patient, thereby impacting how they interact with a traditionally patriarchal medical system. In this context, the ART clinic is used by patients as a tool to bring their own body back in alignment with the life goals they had envisioned for themselves. As a result, those undergoing IVF are active actors who are ‘performing’ the role of the good (and therefore silent) patient not simply because they feel disempowered but rather to further their own goals.

According to Thompson, these performances enact two processes of normalisation. There is the normalising process of ART itself—to ‘return’ women's bodies to the state of being ‘fertile’. But before this can even begin, or so it can continue once it has begun, the individual or couple must meet a set of criteria, both social and biological, that make it likely that they will be good parents. In other words, they need to be the ‘right’ kind of infertile individual or couple. This ‘social’ identity as described by Thompson, is ideally as close to enacting a white, upper middle class nuclear family as possible. To demonstrate that they meet these criteria, the individual or couple seeking ART must ‘stay in control, remain civil, and manifest their emotional and/or relational stability at all times’. The ‘right’ kind of biologically infertile individual is also one who has the potential for successful treatment, which is measured by medicine by markers such as appropriate anatomy, age, body mass index, and anti‐Müllerian hormone levels. Potential patients are asked to modify these measurements where they can, including by losing weight and pre‐emptively freezing their eggs while younger, and thereby admitting them as suitable IVF patients.

Consistent with Katz's view, Thompson suggests patients may wish to avoid breaking character in front of a physician because this expresses an ‘inappropriate’ amount of stress, either about the treatment or its impact on (their) relationship' which ‘indicate(s) unsuitability for and a lack of appreciation of treatment’.^8^ ^,p.93^ However, as postulated by Thompson and reflected in our data, in the ART context both parties are aware that this is a performance, one linked to the patient's desire to become a parent, the doctor's desire to support this, and society's high valuation of fertility.

It is not surprising that our findings from these interviews reflect many of the notions explored by Thompson. A high valuation of family, whiteness, civility, the role of motherhood for a woman, as well as a market economy for the provision of IVF are likely as common in the Australian context as they are in the United States and United Kingdom. However, in the time since Thompson conducted her research, the IVF industry has grown enormously, and with it the IVF patient community. This may have impacted the ways patients and doctors approach discussions around treatment. While the IVF industry has always had commercial backing, the number and power of commercial organisations have grown rapidly over the past decade.^15^, ^16^ At the same time, social media platforms such as Facebook and Instagram and fertility forums are filled with stories about IVF and patients' experiences with various clinicians and clinics.^17^ This includes supportive information about how to manage side effects, how to navigate funding schemes and evaluations of different fertility specialists, both in bedside manner and how willing they were to go straight to further treatment. This is relevant to our results because it suggests that clinicians might over time become feel less of a need to protect ‘informed consumers’ from distressing information, and patients might be increasingly unwilling to tolerate silences on the part of their doctors, or to be silent as they regard themselves as ‘paying customers’. This is evident in the account offered by one of our participants (Consumer 13), who reflected on the impact that living with a chronic illness and being familiar with the health system had on her navigation of IVF. Her familiarity with the role of the patient gave her the confidence to ask questions of her doctor, breaking free from the role of ‘silent’ patient.

It remains to be seen what effect commercialisation and consumer empowerment will have on doctor–patient communication. But it is important not to assume that problematic silences in IVF will simply be eliminated by the transition from a healthcare model to a more transactional service. For example, as more sources of information become accessible to patients outside the clinic, silences about the realities of treatment, at least those described by expert professionals, may grow. At the same time, patients may feel that they should not ask for information because they should already have it as good consumers (and therefore good potential parents). Commercialisation may also exacerbate silences simply because the profit motive discourages extended, time–consumer conversations with patients. Thus, while commercialisation and consumer empowerment may open up discussion, it may also constrain communication and compound silences in the doctor–patient relationship.

### 4.3 Practical implications

Why do the silences described in this paper matter? Most obviously, they matter because consent matters, and because the validity of consent depends on what information is shared between doctor to patient and what each understands. More fundamentally, in Katz's language, the doctor's silences may reflect a distrust and disregard for a patient's decision‐making capacity and even constitute a type of ‘abandonment’, that may not only compromise care but fuel resentment and provoke litigation.

A second reason that silences matter is that if doctors and patients cannot speak openly about the burdens of IVF, then these will remain hidden, thereby compromising their identification, prevention, and management—both in the individual clinical encounter and through more general research and policy initiatives. Taking OHSS as an example, there is clear evidence that women undergoing IVF are either unaware of OHSS and/or do not report it. An article in the Australian Media, March 2022,^18^ reported that ‘One hundred and two women had to spend at least a night in hospital to be treated for ovarian hyperstimulation syndrome (OHSS) across the state [of Victoria] in 2020‐21, an increase of almost 40 per cent from the previous year, according to an audit by the Victorian Assisted Reproductive Treatment Authority (VARTA)’. The Chief Executive of this government regulatory body was then quoted as saying, ‘We want IVF patients to understand what [OHSS] is and when to report symptoms… because a lot of IVF doctors think their own patients don't always report the symptoms of ovarian hyperstimulation…’^18^ A previous IVF patient turned activist who experienced OHSS was also quoted in the article: ‘Now I'm really passionate about raising awareness for ovarian hyperstimulation, because when I was going through IVF, there was nobody else that I knew who talked about it’.^18^

The silences around psychological stresses are likely to be even more pronounced than those around physical side effects. Here, the idea that ‘no one talks about’ the burdens of IVF might also imply that ‘no one should talk about it’. As one of our participants noted, patients feel as ‘they have been sent to the naughty corner’ if they are referred to the counsellor because of their adverse experiences. These feelings translate to action; despite a high level of stress reported by IVF patients, they often do not access counselling services that are available to them.^5^, ^19^ The culture of silence may, therefore, obstruct patients from accessing the services that clinics (in good faith) put in place for them.

So, what should we do about these silences? Here it is helpful to draw on Katz's argument that we need to strike a balance between caring for patients' ‘childlike wishes and needs to be relieved of all responsibility for their care’ while respecting their ‘adult wishes and need to be informed, heard, and consulted’.^7^ ^,p.207^ Crucially, this need to provide a type of care that is supportive of the patient's autonomy is important not just at the first point of contact between patient and health provider, but also at every subsequent encounter throughout the IVF journey. Given the longitudinal nature of these treatments, clinicians need to communicate effectively with patients whose experiences and needs—including for information—may change over time.

A model for this kind of presence and support is offered by Australian empirical bioethicist and humanist Miles Little. While not focused explicitly on IVF, Little articulates five categories of experience that are important in the clinical encounter: *rescue, proximity, ordeal, aftermath* and *presence*.^9^ *Rescue* is the acknowledgement of the power relationship between patient and doctor, which in ART can vary wildly from patient to patient, and even from consultation to consultation. *Proximity* is the acknowledgement of the intimacy of the therapeutic relationship and of medical procedures, which clearly characterise IVF. *Ordeal* is the recognition of the patient's experience of illness and medical interventions. In this regard, it is noteworthy that, in their advertising, corporate IVF clinics downplay the intensity of the treatments they are offering.^20^ *Aftermath* refers to the shift in perception of the self that may occur after medical intervention. This is highly salient in the setting of IVF, as inevitably someone undergoing IVF is left significantly changed—by either a pregnancy or the ongoing ordeal of infertility. Little argues that each of these may be acknowledged and respected through *Presence*—by which he refers to the promise of an ongoing commitment to the patient and a promise not to abandon them irrespective of the outcome of treatment. In the IVF context, this would entail signalling to the patient that even should treatment *never* succeed and they never fall pregnant, the doctor will still want to be in their *presence*. They have value to the doctor as a person, as a patient and not just their potential as a parent.

Putting these suggestions into practice is likely to be challenging for clinicians working in highly competitive commercialised environments, in which there is pressure from management to satisfy as many ‘customers’ as possible, and in which patients might appear to know exactly what they want and need, and even to demand it. The expectations from patients about what clinicians can achieve in IVF is bolstered by the way we as a society talk (or rather don't) talk about IVF in general. Advertising by IVF companies, and social expectations regarding fertility and parenthood may lead patients to not only expect not only a positive outcome, but also to be unprepared for the ordeal of achieving it.

In the face of such forces, clinicians might understandably be tempted to slip into either an entirely consumer‐focused model of care, or an entirely paternalistic one. These temptations must be resisted, and this might be helped by recognising the extent and nature of the silences that characterise the phenomenology of doctor–patient relationships. Recognising what is frequently not said and what is frequently not heard shows that there is enormous scope for improvement in consent, communication and support and that defensive postures—either libertarian or paternalistic—are unnecessary.

## 5 CONCLUSION

The world of ART is currently a silent one. The intentions hidden by these silences leave the burden of unfair treatment on the shoulders of the patient and deprive the doctors of their ability to help them. Changing the way doctors try to be present for patients and the way large IVF providers depict IVF treatment would work in freeing the restrictive expectations both doctors and patients have on themselves.

## CONFLICT OF INTEREST STATEMENT

The authors declare no conflict of interest.

## ETHICS STATEMENT

The study was approved by The University of Sydney Human Research Ethics Committee 2020/320. Participants either gave written consent or had verbal consent recorded at the time of the interview.

## Data Availability

Interview data (recordings or transcripts) will not be made available to maintain participant confidentiality.
